# Supplementary material for: Antisense-mediated repression of SAGA-dependent genes involves the HIR histone chaperone
Source: Nucleic Acids Res. 2022 Apr 26;50(8):4515–28. doi: 10.1093/nar/gkac264 (PMC9071385; doi:10.1093/nar/gkac264)
Supplement: gkac264_Supplemental_Files [file gkac264_supplemental_files.zip › HIR_Supplementary_Figures.pdf]

Figure S1

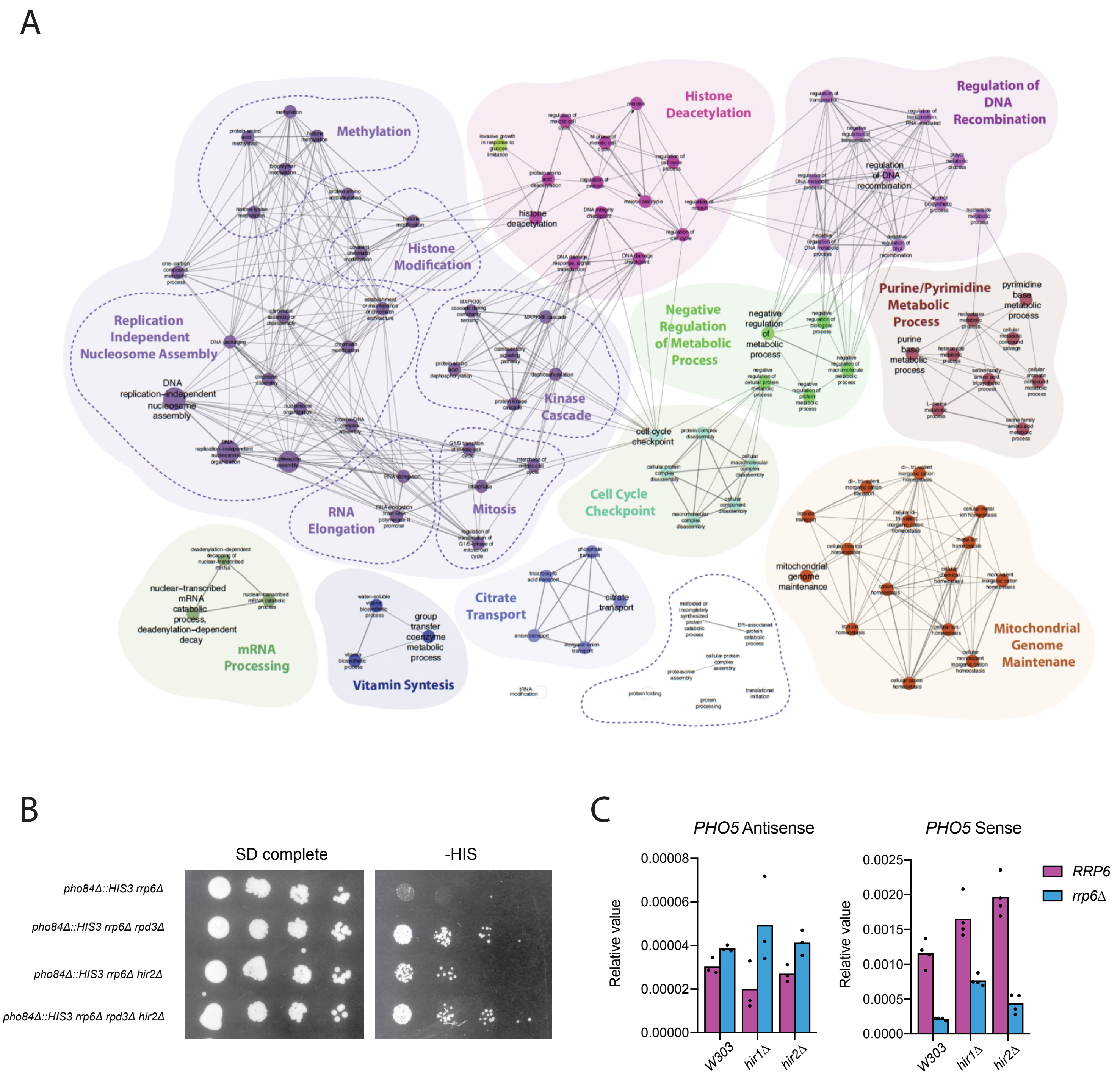

Figure S1: related to Figure 1

(A) ClueGO representation of the genetic screen positive candidates (Cline et al., 2007; Shannon et al., 2003). Each node represents an enriched GO term bearing a minimum of 4 positive candidates. The size of the node reflects the statistical relevance of the GO term. The edges in a ClueGO map indicate that the connected nodes contain proteins sharing the same enriched GO terms.

(B) Growth assay on plates. 10-fold dilutions of the indicated strains were spotted on either SD complete or HIS$^-$ media and grown for 3 days at 25°C.

(C) RT-qPCR analyses of *PHO5* sense and antisense expression normalized to *SCR1* expression in the indicated strains (n=3-4).

Figure S2- related to Figure 2

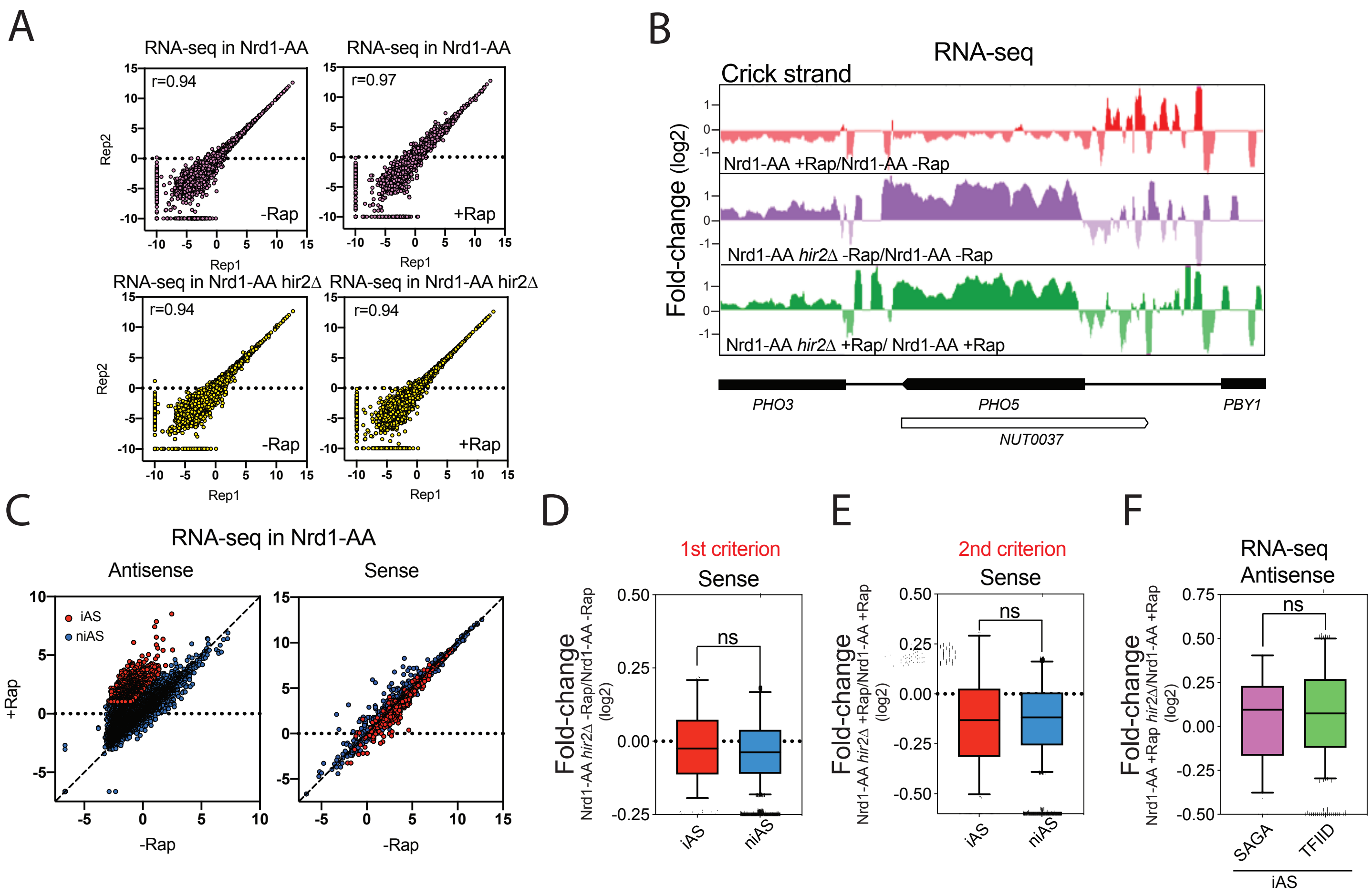

Figure S2: related to Figure 2

(A) Scatter-plot of correlations between RNA-seq replicates. The Spearman correlation coefficient is indicated.

(B) Snapshot of the *PHO5* locus.

(C) Scatter-plot of densities for iAS and niAS in Antisense and Sense orientations. Sense RNAs (TSS to polyA) were considered as one bin giving one value. Antisense RNAs correspond to the same measurement on the other strand.

(D) Boxplot showing the fold-change according to the 1st criterion for the iAS and niAS genes.

(E) Same as in **(D)**, for the 2nd criterion.

(F) Boxplot showing the fold-change (+Rap/-Rap) in Antisense for the iAS SAGA- and TFIID-dependent genes.

Figure S3- related to Figure 3

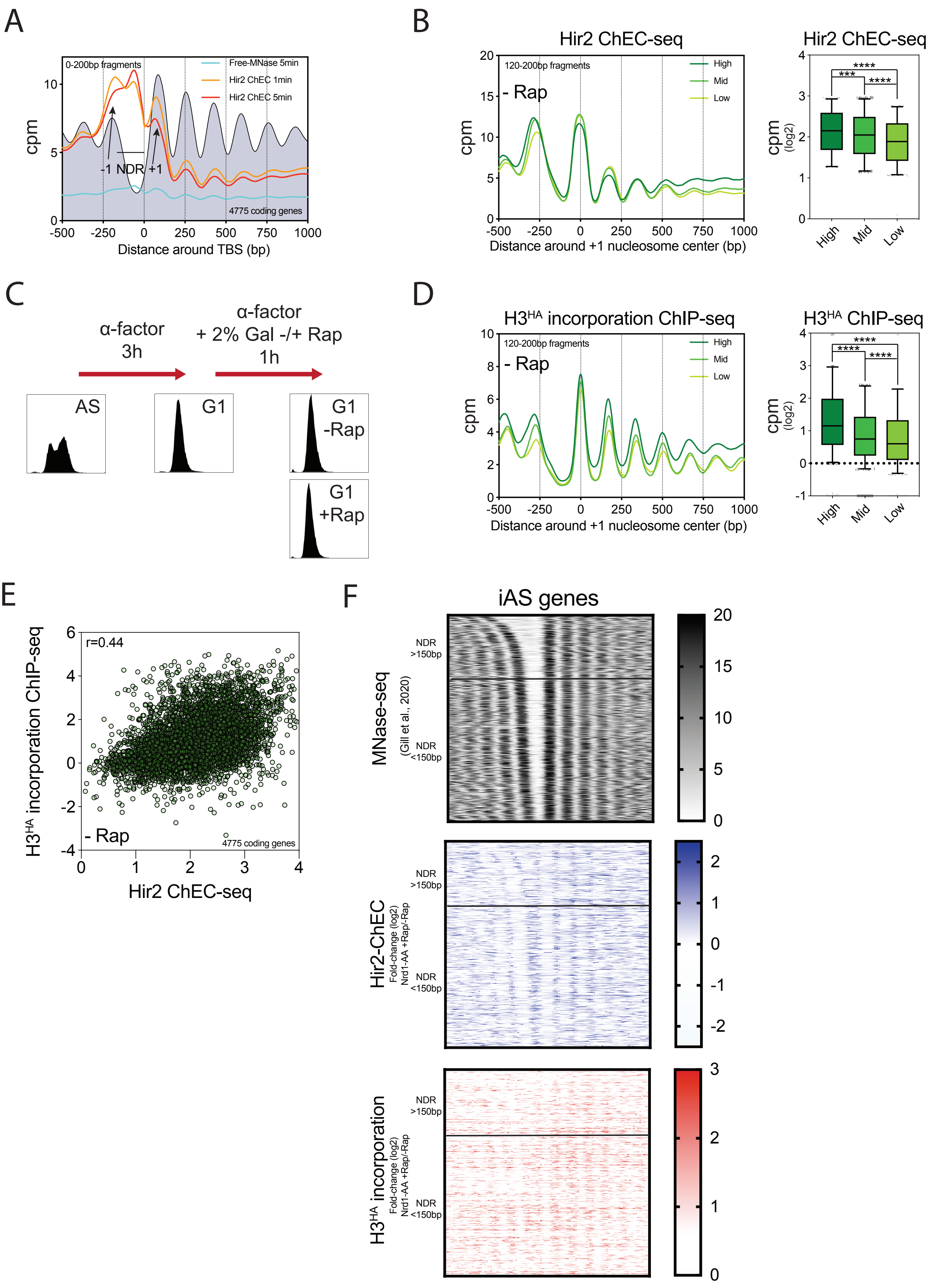

Figure S3: related to Figure 3

(A) Mapping of the Free-MNase and Hir2 ChEC-seq 0-200bp fragments with respect to the TBS of all coding genes (4,775 genes). The dark grey profile depicts the position of nucleosomes as obtained by MNAse-seq.

(B) Left panel: Hir2 ChEC-seq 120-200bp fragments according to the +1 nucleosome center for Highly (1,592 genes), Midly (1,592 genes) and Lowly transcribed genes (1,591 genes). Right panel: Boxplot of Hir2 ChEC-seq signal for Highly, Midly and Lowly transcribed genes considering the whole gene as one bin.

(C) Experimental scheme of the strain culture for the replication-independent $H3^{HA}$ incorporation. Corresponding flow cytometry profiles are indicated.

(D) Same as in **(B)**, with the $H3^{HA}$ incorporation signal.

(E) Scatter-plot showing the correlation between Hir2 ChEC-seq levels and replication-independent $H3^{HA}$ deposition (4,775 genes). The Spearman correlation coefficient is indicated.

(F) Heatmaps of MNase-seq profile in the Nrd1-AA -Rap and of the +Rap/-Rap fold-changes in Hir2 ChEC and newly synthesized $H3^{HA}$ incorporation in the Nrd1-AA strain at the iAS genes. Graphs are centered on the +1 nucleosome center. Each heatmap is divided into two categories of NDR widths: Large NDRs (>150bp) in which a nucleosome can virtually be incorporated, and Small NDRs in which a nucleosome cannot fit.

Figure S4- related to Figure 5

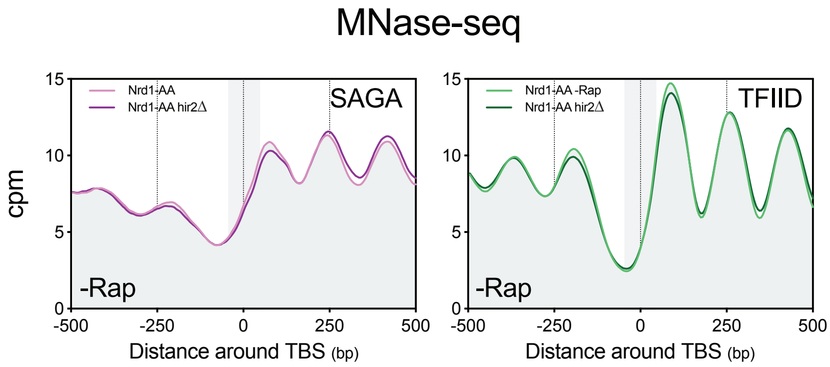

Figure S4: related to the Figure 5

Metagene analyses of the MNase-seq profiles in Nrd1-AA and Nrd1-AA *hir2Δ* strains for SAGA- and TFIID-dependent genes. The grey box represents the 100bp TBS-centered area.

Figure S5- related to Figure 6

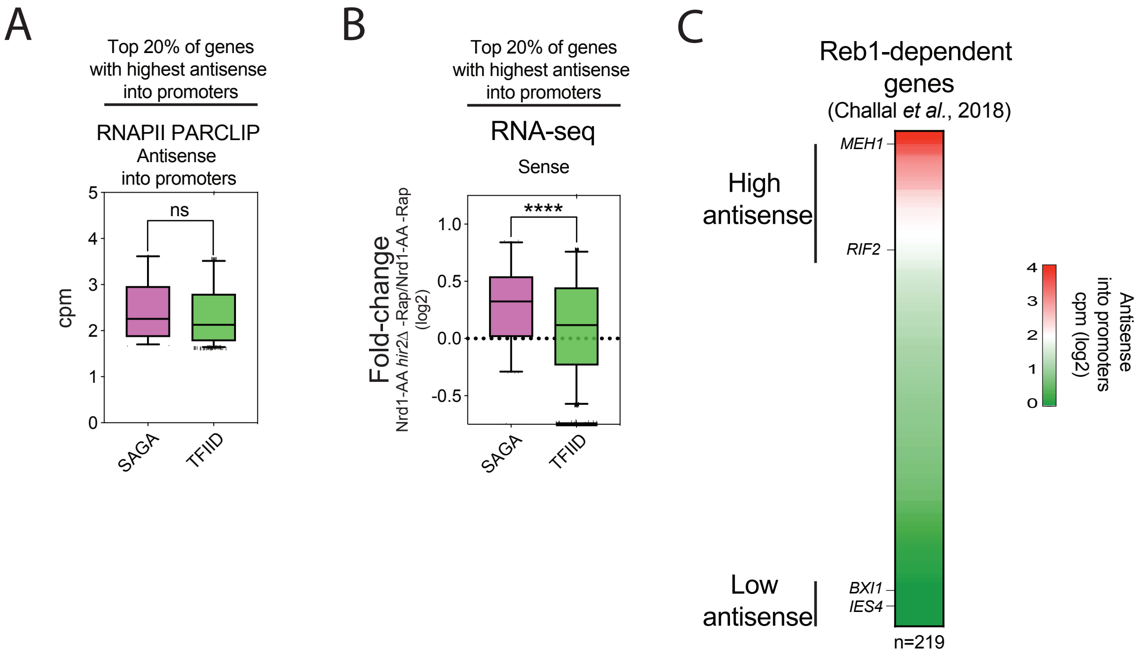

Figure S5: related to Figure 6

(A) Levels of nascent Antisense into promoters (-100 to TSS area) at SAGA- and TFIID-dependent genes for the quintile of genes showing the highest Antisense level into promoters at steady state. A total of 896 genes were examined of which 168 SAGA-dependent and 728 TFIID-dependent. Data were taken from (Schaughency et al., 2014).

(B) Same as in **(A)**, but considering the Nrd1-AA *hir2Δ* -Rap/Nrd1-AA -Rap RNA-seq ratio in Sense orientation.

(C) Heatmap depicting the natural Antisense level into promoters for the Reb1-dependent genes. Genes used in Figure 6C are indicated.

Figure S6- related to Discussion

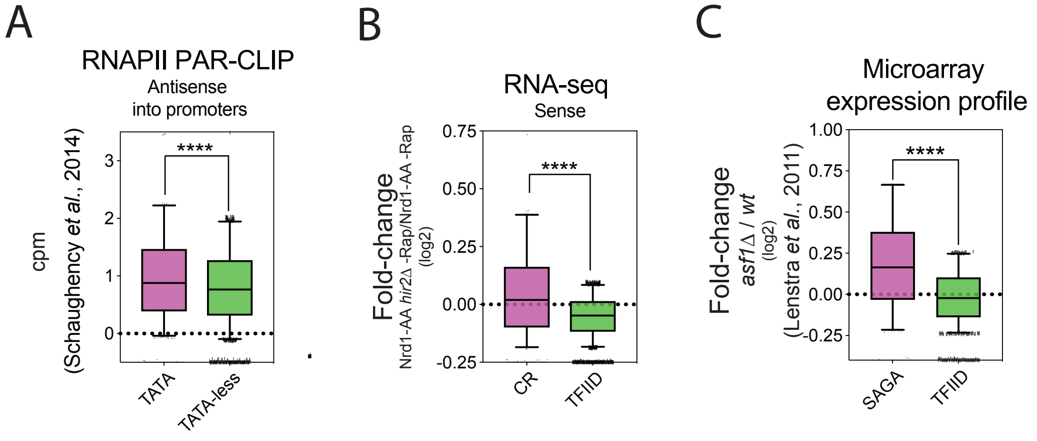

Figure S6: related to the Discussion

(A) Levels of nascent Antisense into promoters (-100 to TSS area) at 649 TATA and 2985 TATA-less dependent genes.

(B) Boxplot depicting the Nrd1-AA *hir2Δ* -Rap/Nrd1-AA -Rap fold-change in Sense of RNA-seq at 523 CR and 3111 TFIID-dependent genes.

(C) Boxplot showing the *asf1Δ/wt* fold-change in Sense expression at the 529 SAGA- and 3967 TFIID-dependent genes. Data were retrieved from (Lenstra et al., 2011).
