# Supplementary material for: Antisense-mediated repression of SAGA-dependent genes involves the HIR histone chaperone
Source: Nucleic Acids Res. 2022 Apr 26;50(8):4515–28. doi: 10.1093/nar/gkac264 (PMC9071385; doi:10.1093/nar/gkac264)
Supplement: gkac264_Supplemental_Files [file gkac264_supplemental_files.zip › TableS_Legends.docx]

**Table S1.** List of the hits from the AMTI genetic screen.

**Table S2.** List of the *S. cerevisiae* strains and primers used in our study.
